# Bayesian spatial analysis of cholangiocarcinoma in Northeast Thailand

**DOI:** 10.1038/s41598-019-50476-7

**Published:** 2019-10-03

**Authors:** Apiporn T. Suwannatrai, Kavin Thinkhamrop, Archie C. A. Clements, Matthew Kelly, Kulwadee Suwannatrai, Bandit Thinkhamrop, Narong Khuntikeo, Darren J. Gray, Kinley Wangdi

**Affiliations:** 1 Department of Parasitology, Faculty of Medicine, Khon Kaen University, Khon Kaen, Thailand; 2 Department of Global Health, Research School of Population Health, College of Health and Medicine, The Australian National University, Canberra, Australian Capital Territory Australia; 3 Data Management and Statistical Analysis Center (DAMASAC), Faculty of Public Health, Khon Kaen University, Khon Kaen, Thailand; 4 Faculty of Health Sciences, Curtin University, Bentley, Western Australia Australia; 5 Department of Biology, Faculty of Science and Technology, Sakon Nakhon Rajabhat University, Sakon Nakhon, Thailand; 6 Cholangiocarcinoma Screening and Care Program (CASCAP), Khon Kaen University, Khon Kaen, Thailand; 7 Department of Surgery, Faculty of Medicine, Khon Kaen University, Khon Kaen, Thailand

**Keywords:** Cancer screening, Cancer epidemiology, Epidemiology

## Abstract

Cholangiocarcinoma (CCA) is a malignant neoplasm of the biliary tract. Thailand reports the highest incidence of CCA in the world. The aim of this study was to map the distribution of CCA and identify spatial disease clusters in Northeast Thailand. Individual-level data of patients with histopathologically confirmed CCA, aggregated at the sub-district level, were obtained from the Cholangiocarcinoma Screening and Care Program (CASCAP) between February 2013 and December 2017. For analysis a multivariate Zero-inflated, Poisson (ZIP) regression model was developed. This model incorporated a conditional autoregressive (CAR) prior structure, with posterior parameters estimated using Bayesian Markov chain Monte Carlo (MCMC) simulation with Gibbs sampling. Covariates included in the models were age, sex, normalized vegetation index (NDVI), and distance to water body. There was a total of 1,299 cases out of 358,981 participants. CCA incidence increased 2.94 fold (95% credible interval [CrI] 2.62–3.31) in patients >60 years as compared to ≤60 years. Males were 2.53 fold (95% CrI: 2.24–2.85) more likely to have CCA when compared to females. CCA decreased with a 1 unit increase of NDVI (Relative Risk =0.06; 95% CrI: 0.01–0.63). When posterior means were mapped spatial clustering was evident after accounting for the model covariates. Age, sex and environmental variables were associated with an increase in the incidence of CCA. When these covariates were included in models the maps of the posterior means of the spatially structured random effects demonstrated evidence of spatial clustering.

## Introduction

Cholangiocarcinoma (CCA) is a malignant neoplasm of the biliary tract and globally is the second most common primary liver cancer^1^. CCA is particularly challenging to address due to it being generally diagnosed late, giving poor survival rates. According to the International Statistical Classification of Diseases and Related Health Problems, 10^th^ revision (ICD-10), CCA comprises: intrahepatic bile duct carcinoma (C22.1), extrahepatic bile duct carcinoma (C24.0), and ampulla of Vater carcinoma (C24.1). Incidence rates for CCA vary widely from around 0.2 per 100,000 population in Australia to 96 per 100,000 in men in Northeast Thailand^2^. Overall the largest burdens for this cancer are found in and Southeast Asia^3^. Within Northeast Thailand itself, which has the world’s highest rates of CCA, age-standardized incidence varies. For example, rates ranging between 93.8 to 317.6 per 100,000 person-years between villages in Khon Kaen Province from 1990 to 2001^4^. This variation in incidence rates is most likely due to differences in environmental and genetic factors that affect the disease and its aetiology^5^.

There are several recognized aetiological agents for CCA including infection with *Opisthorchis viverrini*, a hepatobiliary fluke. This infection has been identified as the leading cause of CCA in Southeast Asia, including Northeast Thailand^6^. The International Agency for Research on Cancer within the World Health Organization (WHO), classifies *O. viverrini* as a Group 1 human carcinogen^7^. This liver fluke is widely distributed in Thailand, Lao People’s Democratic Republic (PDR), Cambodia, Vietnam and Myanmar. In Lao PDR and Thailand an estimated 10 million people are infected^8,9^. Infection with *O. viverrini* is acquired by eating raw or insufficiently cooked cyprinid fish containing *O. viverrini* metacercariae. Once ingestion occurs, *O. viverrini* commonly invade the bile ducts and cause pathological changes to the bile ducts, liver and gall bladder^6^. Infection with *O. viverrini* has been recognized as leading to several hepatobiliary diseases. These include cholangitis, obstructive jaundice, hepatomegaly, fibrosis of the periportal system, cholecystitis, and cholelithiasis and is a major aetiological agent of CCA^10,11^. Chronic *O.viverrini* infections lead to bile duct inflammation, inducing oxidative and nitrative damages of tissues and DNA, which may finally progress to CCA and death^6,12–14^. The intermediate host of the *O.viverrini* liver fluke is the *Bithynia* snail and environmental suitability for this snail host is important for the ability of the *O. viverrini* life cycle to continue.

To gain a more comprehensive understanding of the distribution of CCA and its determinants it is important to assess the geographic and environmental factors that may help explain the distribution. Geographical information system (GIS), remote sensing (RS) and spatial Bayesian statistical methods are tools that have been used for mapping cancers^15–17^ and diseases with an infectious origin^18–20^ that are strongly influenced by environmental characteristics including climate. The outcomes of such mapping exercises can help inform the design of large-scale disease control programmes such as is required to address CCA in Thailand. In this study, we aimed to map the distribution and identify spatial clusters of CCA in Northeast Thailand.

## Results

### Descriptive statistics

**Figure 1:**
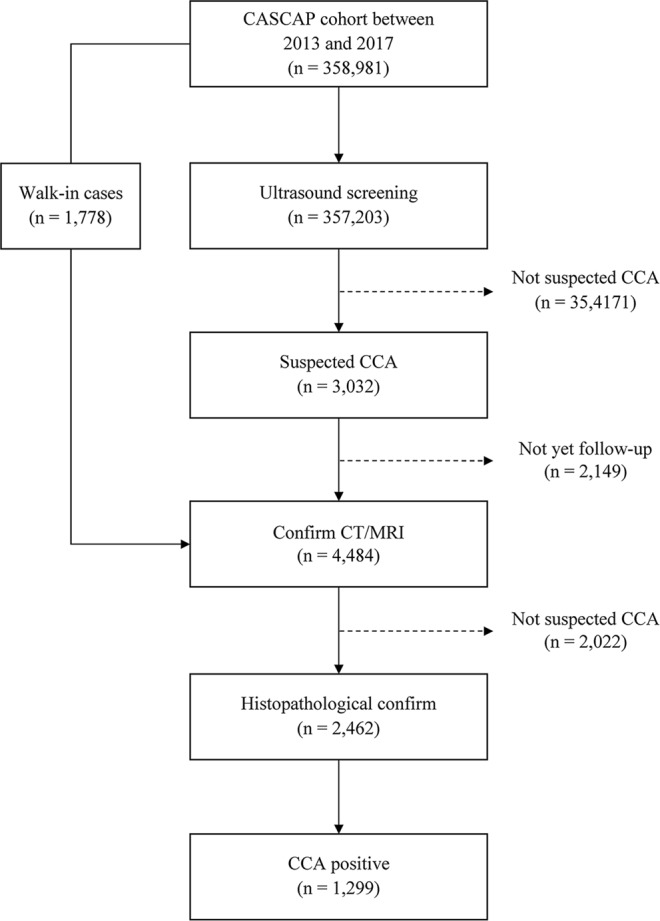
Study participants and CCA cases.

Table 1 and Fig. 1 describe the study participants. There were 358,981 subjects enrolled in the CASCAP project between 2013 and 2017. Of these, approximately two-third (61.2%, 219,666) were female and around one-quarter (26.2%, 93,478) were aged >60 years, with a mean age of 54.49 (SD = 9.47) years. Overall, 1,299 participants or 0.36% of the cohort were diagnosed as CCA cases. The CCA cases were more common among males than females (0.6% and 0.21%, respectively). Around 0.73% (683) of the CCA cases were >60 years old. In stratified analysis, CCA cases were most common among males aged more than 60 years old, at 1.06% (451/42,635) (Table 2).

**Table 1:** Percentage of cholangiocarcinoma cases according to sex and age groups.

Variables	Participants (%)	Number of CCA cases	Percentage (95% CI)
Over all	358,981	1,299	0.36 (0.34–0.38)
**Sex**			
Female	219,666 (61.20)	461	0.21 (0.19–0.23)
Male	139,274 (38.80)	838	0.60 (0.56–0.64)
**Age**			
≤60	262,883 (73.80)	616	0.23 (0.22–0.25)
>60	93,478 (26.2)	683	0.73 (0.68–0.79)

**Table 2:** Cholangiocarcinoma cases stratified by age and sex.

Sex	Age in years	Participants	Number of CCA	Percentage (95% CI)
Female	≤60	167,200	229	0.14 (0.12–0.15)
	>60	50,841	232	0.46 (0.40–0.51)
Male	≤60	95,670	387	0.40 (0.36–0.44)
	>60	42,635	451	1.06 (0.96–1.15)

**Figure 2:**
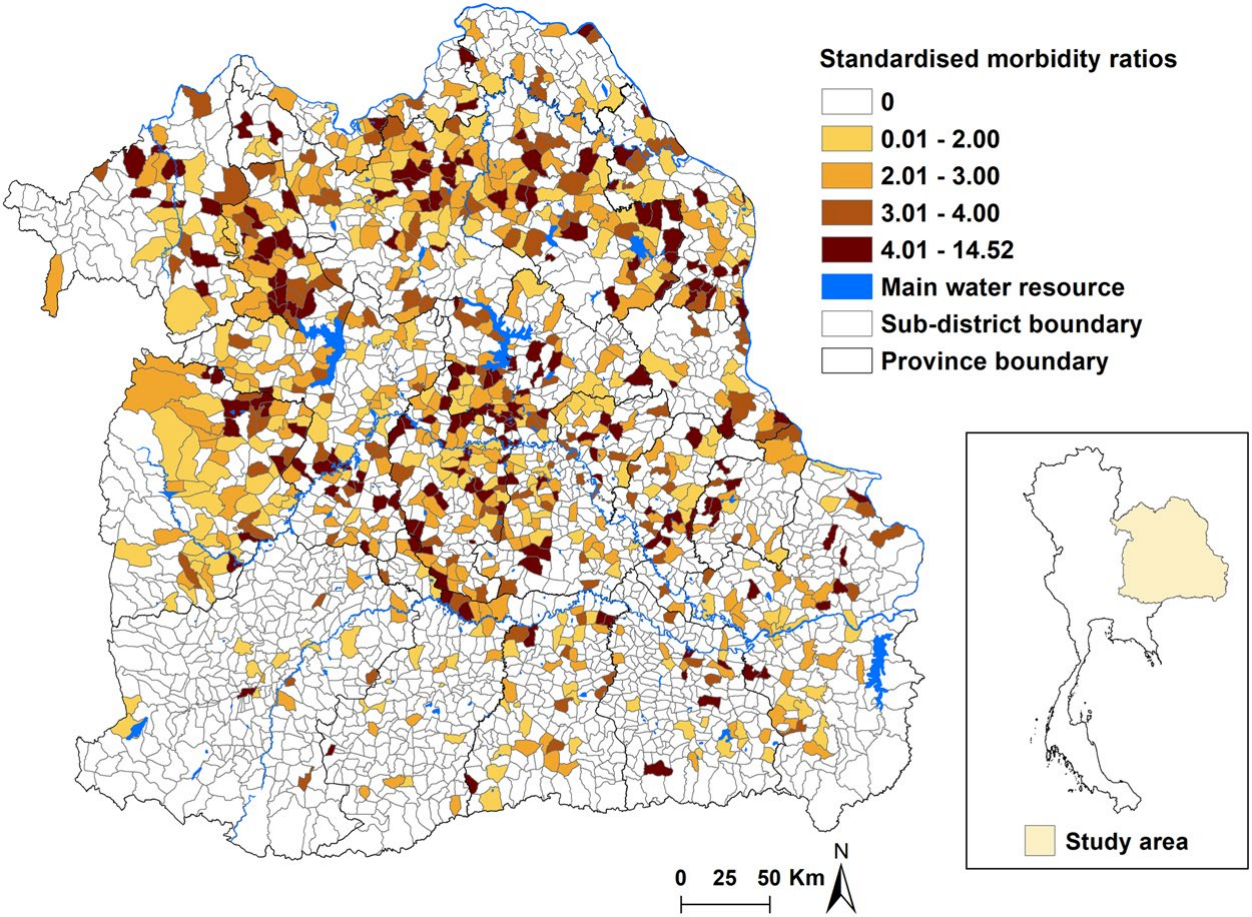
Map of the raw standardized morbidity ratios (SMRs) for cholangiocarcinoma across sub-district level in Northeast Thailand, 2013–2017.

The overall crude incidence rate of CCA for the five-year period was 2.64 cases per 100,000 population, with sub-district rates ranging from 0 to 38 cases per 100,000 population. Strong spatial variations were observed in the CCA incidence rate across sub-districts. Figure 2 shows the spatial distribution of the SMRs for CCA in Northeast Thailand at the sub-district level.

### Spatial poisson regression analysis

**Figure 3:**
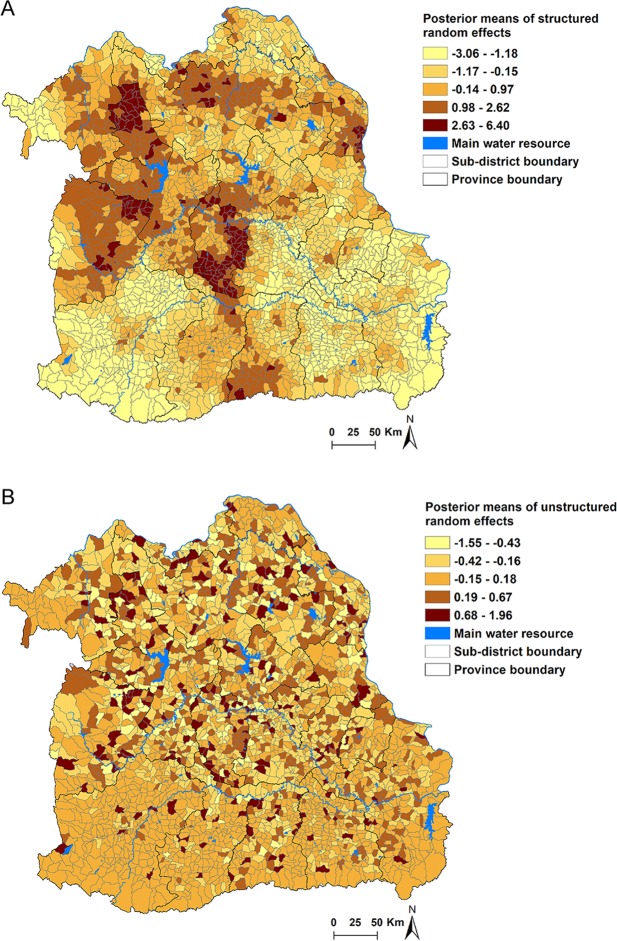
Spatial distributions of the posterior means of random effects for cholangiocarcinoma in Northeast Thailand in Model III. (**A**) Spatially structured random effects (**B**) unstructured random effects.

The Bayesian spatial and non-spatial models for CCA are presented in Table 3. Using the DIC, the CCA model III with both unstructured and structured random effects was the best-fitting model. In this best-fit model (Model III), age, sex, and NDVI were significantly associated with incidence of CCA. Individuals aged >60 years were found to be 2.94 times (95% CrI: 2.62–3.31) more likely to have CCA than those aged ≤60 years, and males were 2.53 times (95% CrI: 2.24–2.85) more likely to have CCA when compared to females. NDVI was negatively associated with CCA incidence. There was an estimated decrease of 94% (95% CrI: 37%-99%) of CCA cases for 1-unit increase in NDVI. The map of the posterior means of the spatially structured random effects demonstrated evidence of spatial clustering after accounting for the model covariates (Fig. 3). A large high-risk area cluster of CCA was found in Maha Sarakham Province, which is located in the Chi and Mun River Basin. More circumscribed areas of high risk of CCA were observed in Nong Bua Lamphu, Khon Kaen and Chiyaphum Provinces. There were more isolated high risk sub-districts found in Nakhon Phanom, Sakon Nakhon and Udon Thani Provinces, which are located in the Mekong River Basin (Fig. 3A) (see Supplementary file). The unstructured random effects showed a random spatial pattern as expected (Fig. 3B).

**Table 3:** Regression coefficients, RRs and 95% CrI from Bayesian spatial and non-spatial models for cholangiocarcinoma in Northeast Thailand. Note. ^*^Age ≤ 60 years was reference. ^**^Female sex was reference. ^***^Best fit model. Key: CrI = credible intervals; RR = relative risks; DIC = deviance information criterion.

Model/variables	Coefficient, posterior mean (95% CrI)	RR, posterior mean (95% CrI)
**Model I (Unstructured)**		
α (Intercept)	−7.21 (−7.39–7.04)	
Age*	1.08 (0.97–1.20)	2.96 (2.62–3.32)
Sex**	0.93 (0.81–1.05)	2.54 (2.25–2.87)
NDVI (Unit)	−0.30 (−0.43–0.17)	0.02 (0.004–0.11)
Distance to water body (Km)	−0.20 (−0.34–0.06)	0.82 (0.72–0.94)
Heterogeneity		
Structured (variance)	—	—
Unstructured (variance)	0.22 (0.19–0.26)	—
DIC	7198.44	
**Model II (Structured)**		
α (Intercept)	−7.14 (−7.30–6.98)	
Age*	1.08 (0.96–1.19)	2.93 (2.61–3.29)
Sex**	0.93 (0.81–1.05)	2.53 (2.25–2.85)
NDVI (Unit)	−0.19 (−0.38–0.01)	0.10 (0.01–1.16)
Distance to water body (Km)	−0.02 (−0.16–0.12)	0.98 (0.86–1.12)
Heterogeneity		
Structured (variance)	0.08 (0.07–0.09)	—
Unstructured (variance)	—	—
DIC	7051.19	
**Model III (Structured and unstructured)**		
α (Intercept)	−7.15 (−7.33–6.99)	
Age*	1.08 (0.96–1.20)	2.94 (2.62–3.31)
Sex**	0.93 (0.81–1.05)	2.53 (2.24–2.85)
NDVI (Unit)	−0.22 (−0.41–0.04)	0.06 (0.01–0.63)
Distance to water body (Km)	−0.02 (−0.16–0.11)	0.98 (0.86–1.12)
Heterogeneity		
Structured (variance)	0.12 (0.10–0.17)	—
Unstructured (variance)	1.00 (0.63–1.72)	—
DIC	7016.86***

## Discussion

The current study represents a spatial analysis of the largest CCA screening program carried out to date, and which is part of the planning activities of a nation-wide opisthorchiasis and CCA control programme. We found that CCA was geographically clustered in Maha Sarakham Province. Sub-District characteristics associated with higher rates of CCA were identified including the demographic profile and NDVI.

We used data on CCA cases identified by the CASCAP program between 2013 and 2017. This data source is ideal for a number of reasons. Firstly, the CASCAP program adopts an active recruitment and screening process. This means CASCAP is able to identify CCA cases at an early stage of progression. Secondly, the CASCAP project uses the complete range of confirmatory diagnosis methods (ultrasound, MRI, CT and histopathology). This means the CASCAP data have high reliability and validity. Finally, the CASCAP project is designed as a cohort study. Those identified as at risk of developing CCA were actively followed and received regular screening giving us a more reliable source of information on CCA progression^42^.

CCA cases had positive associations with demographic variables in terms of increasing age and being male. Many previous research studies conducted in Thailand have also reported that CCA is common among older age groups and males^6,21–23^. Recently, Chaiteerakij *et al*. (2017) utilized the National Hospital Admission Data registry to identify the characteristics of CCA patients between 5 different regions of Thailand in the period 2008 and 2013. In this study, similar to our results, CCA patients in Thailand had a mean age of 64.0 ± 11.7 years and 61.2% were males. The greatest number of CCA cases (24,239; 61.5%) was found in the Northeast region^23^. This finding is also similar to previous research reported by Haswell-Elkins *et al*., (1994) which identified older aged participants (>50 years old) as having 9.21 times the odds of having CCA when compared with younger age groups (confidence interval [CI] (1.10–77.11)^21,24^. The exact reasons for the increased CCA risk in older age in this region are not yet fully understood. Many factors were reported such as chronic inflammation, accumulated genetic alterations, immune response and cumulative past exposure to *O. viverrini* infection^6,25–27^. The patterns of infection with *O. viverrini* in Northeast Thailand assessed by egg count, parasite-specific antibody levels and worm burden increased significantly with age^28^. This finding shows that the infection began at an early age. In endemic areas where there is continuous infection, age is a quantitative representation of the length of time over which liver fluke infections can occur. Males were also 3.00 times (OR 3.00; 95% CI 0.80–11.25) more likely to develop CCA than females^21,22,24^. Males appear more likely to incur *O. viverrini* infection than females, due to their behavior related to eating raw cyprinid fish, smoking behavior, alcohol consumption as well as genetics^27,29^, and this then leads to higher CCA rates.

We also found that risk of CCA was negatively associated with NDVI, suggesting that NDVI can explain some of the spatial distribution of CCA. The impact of NDVI on CCA is likely to relate to the environmental factors that affect snail habitats^30,31^. The first intermediate host for *O. viverrini* are *Bithynia* sp. snails. These snails release cercariae into water sources facilitating transmission to cyprinid fish, which harbor the infective stage to humans (metacercaria). Infection in humans then is influenced by a range of factors including the density and distribution of snail populations. These factors include the likelihood of infected human and reservoir species faeces reaching the water body, and the likely survival and success of the free swimming cercariae that exit the snail, attach and penetrate into the tissue of cyprinid fish, determine the potential infective dose to humans^8,32^.

Snail survival appears to be negatively associated with NDVI; as the NDVI increases, snail survival probability decreases^33^. Recent studies by Pratumchart and colleagues (2019) used Maximum Entropy (MaxEnt) algorithm software to generate predictive risk map of distribution pattern for *B. s. goniomphalos* in relation to climatic and environmental factors in Northeast Thailand. The study found that the distribution of *B. s. goniomphalos* was negatively correlated with NDVI^34^. The dominant habitat type of *B. s. goniomphalos* is rice paddy fields^35^. In Northeast Thailand, paddy fields are the most common type of land cover with patches of remnant forests, which can either evergreen and deciduous forests. The paddy fields represent low NDVI as opposed to dry evergreen forests (highest score)^36^. Smaller snail populations would reduce the risk of *O. viverrini* infection and resulting CCA, these results help to explain the relationships found in our study. In addition, the transmission dymanic of *O. viverrini* infection mostly occurred in rice paddy fields where the two intermediate hosts of *O. viverrini* are abundant and local inhabitants, and where people get infections^37–41^.

Significant clusters of CCA were located in Maha Sarakham, Nong Bua Lamphu Khon Kaen, Chiyaphum, Nakhon Phanom, Sakon Nakhon and Udon Thani Provinces. These areas lie in the basins of the Chi and Mekong Rivers, where the high prevalence of opisthorchiasis and cholangiocarcinoma have been documented^6,42^. Our study was undertaken using co-variates that mainly focused on environmental factors including NDVI, NDWI, LST and altitude. However, future studies should include other important factors such as soil type, soil texture and land cover that could further explain spatial variation in CCA risk.

## Conclusions

This work demonstrates that older (>60 years), male individuals were at a higher risk of CCA than younger individuals and females. NDVI was associated with a reduced risk of CCA, likely related to vegetation type (with low-NDVI rice paddy areas having the highest risk). The maps of the posterior means of the spatially structured random effects of CCA demonstrated evidence of spatial clustering after accounting for the model covariates. These results can help inform the current Thai government CCA control program by allowing appropriate targeting of resources to surveillance, CCA treatment and preventive interventions targeting the *O. viverrini* parasite.

## Methods

### Study area

**Figure 4:**
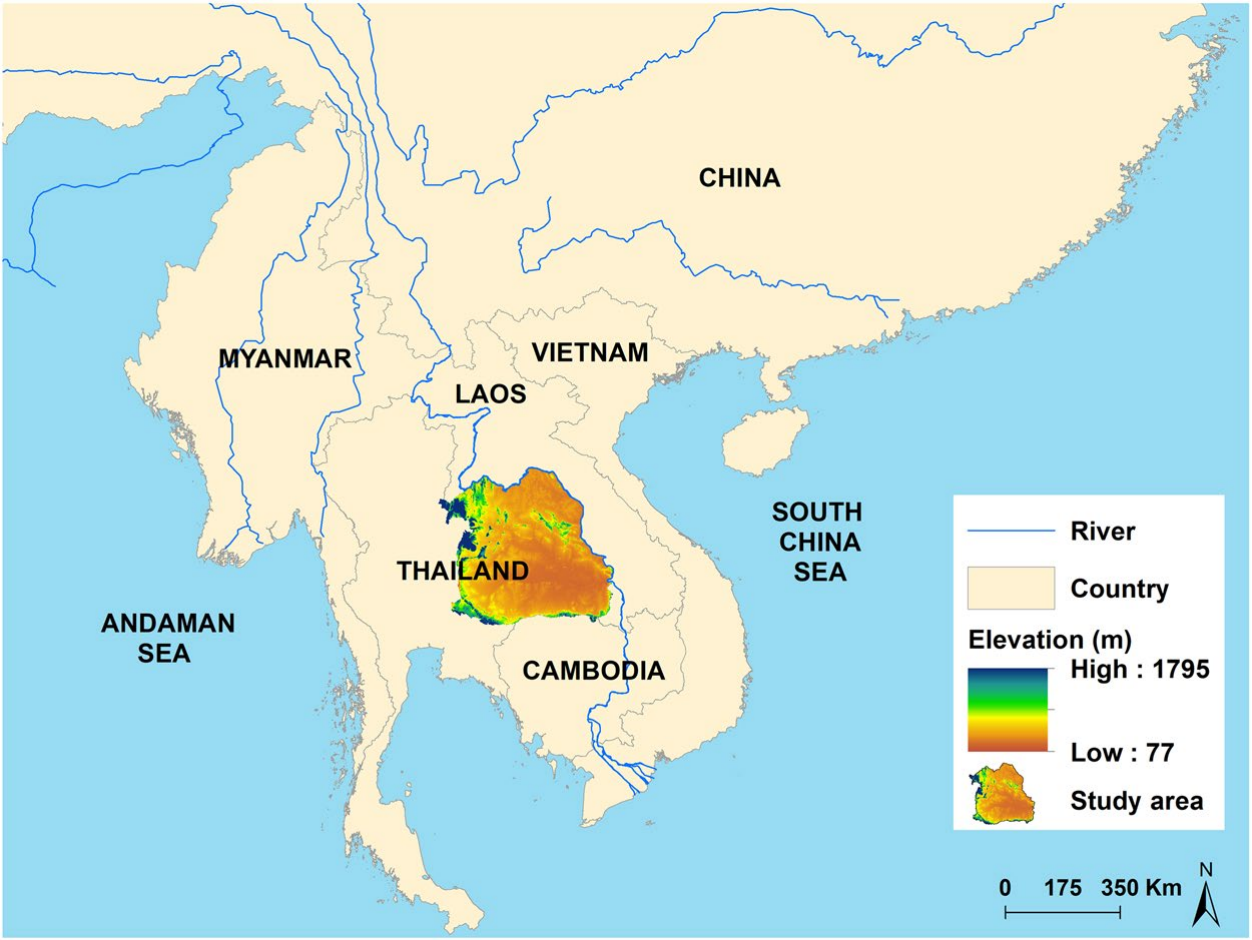
*Map of Northeast Thailand and neighboring countries.*

The study was undertaken in Northeast Thailand, which is located between latitudes 14.50°N and 17.50°N, and between longitudes 102.12°E and 104.90°E and which covers an area of approximately 168,854 km^2^(Fig. 4). This study investigates CCA distribution in all 2,678 sub-districts in all 20 provinces of Northeast Thailand, with a population of 22.24 million in 2018.

### Study participants

The Cholangiocarcinoma Screening and Care Program (CASCAP) is a prospective cohort study undertaken by the Faculty of Medicine, Khon Kaen University, in cooperation with the Cholangiocarcinoma Foundation, Thailand. CASCAP program activities comprise of a prevention and patient care program among at-risk populations in high-risk areas for CCA. The three stage program includes: (1) opisthorchiasis screening, (2) CCA and periductal fibrosis screening, and (3) confirmatory diagnosis of suspected CCA patients, plus treatment and care^43^. Detailed recruitment procedures have been published elsewhere, but in summary, participants are enrolled actively through primary health care units if they fit the inclusion criteria, or are self-enrolled when they perceive risk of CCA and attend a health facility^44^.

CASCAP participants were enrolled in the CCA and periductal fibrosis screening stage of the program if they met the following criteria: (1) reside in Northeast Thailand, (2) aged ≥40 years, (3) past history of *O. viverrini* infection, (4) past history of treatment for *O. viverrini* infection with praziquantel, and (5) past history of consumption of raw or undercooked cyprinid fish^44,45^. The individuals at risk for *O. viverrini*-induced CCA underwent a liver ultrasound examination to determine their bile duct or liver pathology. Those suspected of CCA by ultrasound or clinical symptoms were further subjected to a confirmatory test/diagnosis using magnetic resonance imaging (MRI) or computerized tomography (CT) scanning and histopathological diagnosis to confirm CCA^44^. The patients with CCA were offered treatment and follow up care.

The study population then for this study was all CASCAP participants who received histopathological confirmation of CCA diagnosis between February 2013 and December 2017. Individual-level data for these patients were aggregated at the sub-district level, based on the CASCAP database. Sub-district populations for the study period (2013–2017) were obtained from the Official Statistics Registration Systems of Thailand website (http://stat.bora.dopa.go.th/new_stat/webPage/statByYear.php). For this analysis, CCA cases were stratified into two age groups reflecting CCA risk: ≤60 years and >60 years.

### Environmental data

MODIS data products including land surface temperature (LST), normalized difference vegetation index (NDVI) and normalized difference water index (NDWI) were downloaded from the United States Geological Survey (USGS) EROS Data Center. Environmental data were collected for 1 January 2001 through 31 December 2017. Resolution was set at 0.25 km^2^ and data were processed to produce a mosaic covering the study area in Thailand using ArcGIS 10.5.1 (ESRI Inc., Redlands, CA, USA). Altitude data were obtained from the WorldClim database (www.worldclim.org). Polyline shapefiles for streams and rivers and a polygon shapefile for administrative boundaries at the sub-district level of Northeast Thailand were obtained from the DIVA-GIS website (www.diva-gis.org). The administrative boundary map included 2,678 sub-district level areas.

Spatial data including CCA cases, demographic and environmental data, were imported into the ArcGIS 10.5.1 and projected to the Universal Transverse Mercator (UTM) coordinate system zone 48 N. The datasets were linked according to the location of the administrative boundary map of Northeast Thailand to summarize and extract the data by sub-district area and define parameters for subsequent statistical analyses.

## Data Analysis

### CCA incidence rate

Firstly, a descriptive analysis of CCA cases was performed. The crude incidence rate of CCA was calculated by dividing the number of CCA cases recorded in the five-year study period (2013–2017) by the total population during the same time period. The Standardized Morbidity Ratios (SMR) for CCA were calculated for each sub-district in Northeast Thailand for 2013–2017 using the formula:

$${Y}_{i}=\frac{{O}_{i}}{{E}_{i}}$$

In this formula where *Y* represents the SMR in sub-district *i*, *O* the reported number of CCA cases in the sub-district and *E* the expected number of CCA cases occurring in that sub-district during the study period. To estimate the expected number of CCA cases for each sub-district the population of each sub-district was multiplied by the overall crude CCA incidence rate for the study area and period^46^.

### Variable selection

A preliminary univariate Poisson regression of CCA case numbers was undertaken with age, sex, NDVI, NDWI, LST, altitude, and distance to water body to examine associations with CCA incidence. Age, sex, NDVI and distance from the water body were the only significant variables (p < 0.05) in the univariate models and were selected for the final models based on them having the lowest Bayesian information criterion (BIC)^19,47^. Finally, Pearson correlation analyses were conducted to assess collinearity among all the included variables. All the preliminary statistical analyses were performed using the STATA software version 14.0 (Stata Corporation, College Station, TX, USA).

### Spatial poisson regression analysis

Zero-inflated Poisson (ZIP) regression models were built using a Bayesian framework in the WinBUGS software, version 1.4.3 (Medical Research Council, Cambridge, UK). Explanatory variables included in the first model (Model I) were age (≤60 years and >60 years), sex, NDVI and distance to water body. An unstructured random effect at the sub-district level was also included. The same explanatory variables were included in the second model (Model II) as well as a spatially structured random effect. The final model (Model III), a convolution model, included all of the variables of the preceding two models.

The convoluted model, with an outcome of observed cases of CCA (numbers), *Y*, for *i*^th^ sub-district in Northeast Thailand (*i* = 1 to 2678), for the *k*^th^ age group and *l*^th^ sex group was structured as follows:

$$\begin{array}{rcl}{Y}_{ikl} & \sim  & {\rm{Poisson}}\,({\mu }_{ikl})\\ \log ({\mu }_{ikl}) & = & \log ({{\rm{E}}}_{ikl})+{\theta }_{ikl}\end{array}$$

*θ*_*ikl*_ = *α* + *β*_1_ × age_*k*_ + *β*_2_ × sex_*l*_ + *β*_3_ × NDVI_*i*_ + *β*_4_ × distance to water body_*i*_ + u_*i*_ + s_*i*_ where E_*ikl*_ is the expected number of CCA cases (offsetting population size) in sub-district *i*, age-group *k*, sex group *l* and θ_*ikl*_ is the mean log relative risk (RR); α is the intercept, and *β*_1_*, β*_2_*, β*_3_, and *β*_4_ the coefficients for age (≤60 years as the reference category), sex (female as the reference category), NDVI, and distance to water body; u_*i*_ is the unstructured random effect (assumed to have a mean of zero and variance σ_u_^2^) and s_*i*_ is the spatially structured random effect (assumed to have a mean of zero and variance σ_s_^2^).

In modelling the spatially structured random effects, a conditional autoregressive (CAR) prior structure was used. An adjacency weights matrix was then used to assess spatial relationships between sub-districts. Where two sub-districts shared a border, a weight of 1 was assigned, otherwise, the weight was 0. The intercept was defined utilizing a flat prior distribution. For the coefficients a normal prior distribution was used. A mean of zero was set, and precision, the inverse of variance, was set at 0.0001. Non-informative gamma distribution equal to 0.001 was used as The priors for the precision of unstructured and spatially structured random effects.

**Figure 5:**
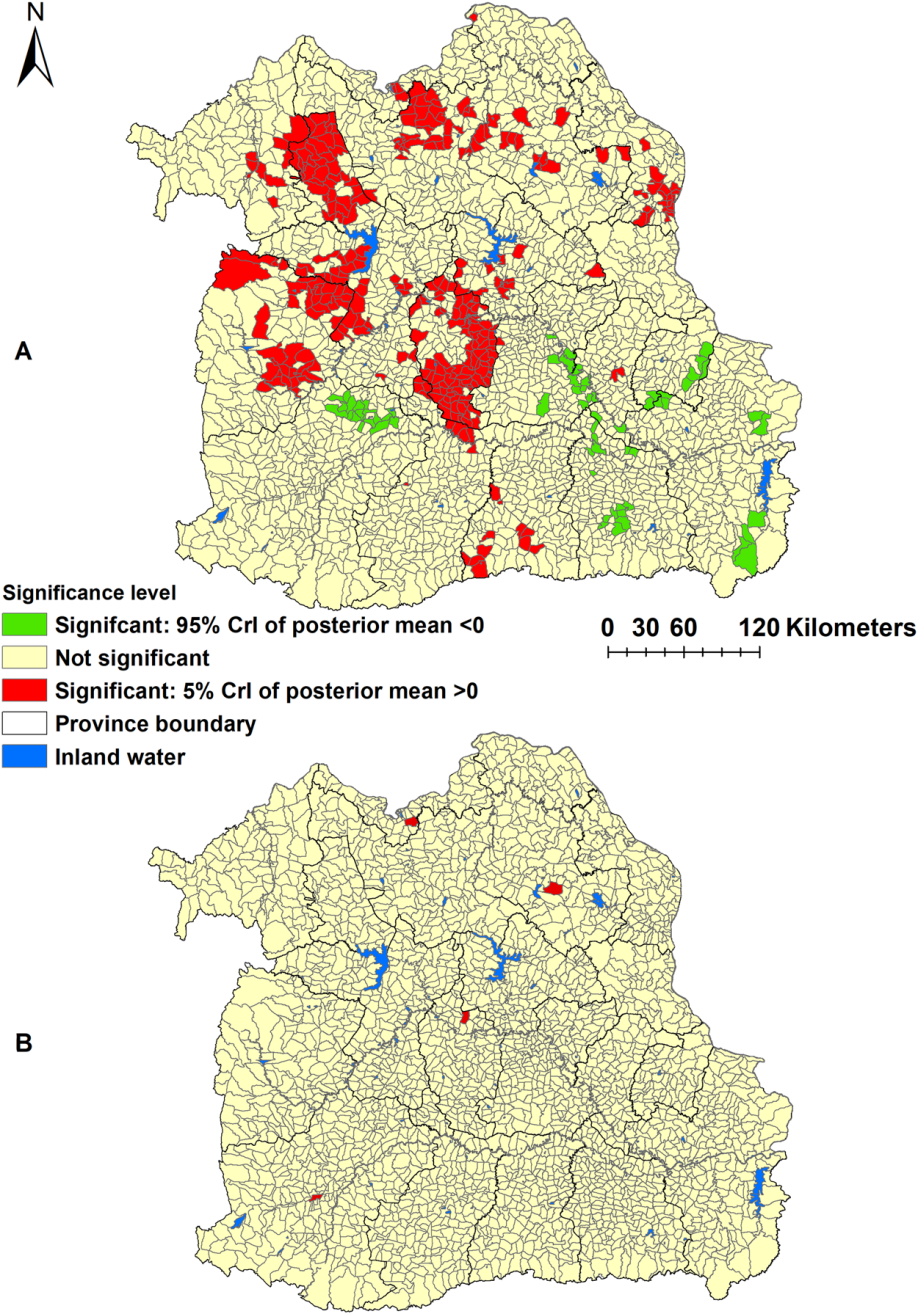
*Significance map of posterior means of random effects of cholangiocarcinoma in Northeast Thaila*n*d*. (**A**) Spatially structured random effects (**B**) unstructured random effects.

The model was run for an initial burn-in of 10,000 iterations and discarded. Then subsequent sets of 15,000 iterations were run with Monte Carlo chains until convergence was achieved. A total of three sets of 15,000 iterations were run (and discarded) before convergence was achieved as assessed by visual examination of posterior density plots and confirmed by Gelman-Rubin statistics. The best fit model was selected based on the lowest deviance information criterion (DIC), more parsimonious model. Statistical significance at an α-level of 0.05 (calcualted by 95% credible intervals (95% CrI) for relative risks (RR) that excluded 1) was used for this study^47^. ArcGIS 10.5.1 software was used to generate maps of the spatial distribution of posterior means of the unstructured and structured random effects. The significance level of posterior random means were calculated using the using 5% and 95% CrI of posterior random effects and a significance map was created (Fig. 5).

### Ethics statement

The Khon Kaen University Ethics Committee for Human Research approved this study (HE611035). The CASCAP was conducted according to the principles of Good Clinical Practice, the Declaration of Helsinki, and national laws and regulations about clinical studies, and was approved by the Khon Kaen University Ethics Committee for Human Research under the reference number HE551404. All patients gave written informed consent for the CASCAP study.

## Supplementary information

Supplementary Figure 1

## Data Availability

The datasets generated during and/or analyzed for the current study will be made available from the corresponding author on reasonable request.
