# Supplementary material for: Bayesian spatial analysis of cholangiocarcinoma in Northeast Thailand
Source: Sci Rep. 2019 Oct 3;9:14263. doi: 10.1038/s41598-019-50476-7 (PMC6776517; doi:10.1038/s41598-019-50476-7)
Supplement: Supplementary file 1 — Supplementary Figure 1 [file 41598_2019_50476_MOESM1_ESM.pdf]

Bayesian spatial analysis of cholangiocarcinoma in Northeast Thailand

Apiporn T. Suwannatrai, Kavin Thinkhamrop, Archie C. A. Clements, Matthew Kelly, Kulwadee Suwannatrai, Bandit Thinkhamrop, Narong Khuntikeo, Darren J. Gray & Kinley Wangdi

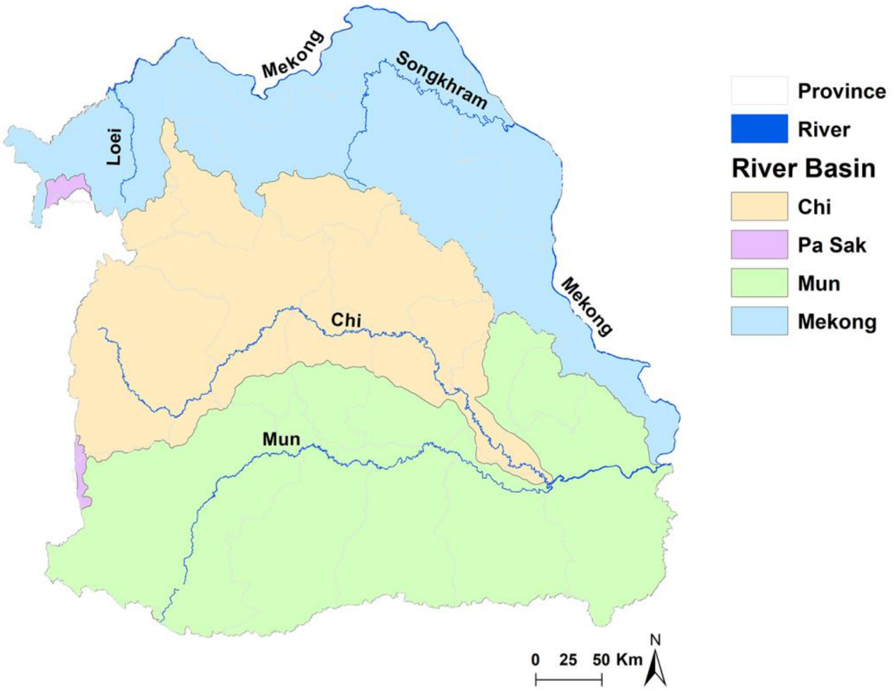

Supplementary Figure 1. Map of provinces, rivers, and river basins in Northeast Thailand.
